# DNA Vaccines: MHC II-Targeted Vaccine Protein Produced by Transfected Muscle Fibres Induces a Local Inflammatory Cell Infiltrate in Mice

**DOI:** 10.1371/journal.pone.0108069

**Published:** 2014-10-09

**Authors:** Tom-Ole Løvås, Jo C. Bruusgaard, Inger Øynebråten, Kristian Gundersen, Bjarne Bogen

**Affiliations:** 1 Department of Immunology, University of Oslo and Oslo University Hospital, Rikshospitalet, Oslo, Norway; 2 Centre for Immune Regulation, University of Oslo, Oslo, Norway; 3 Department of Biosciences, University of Oslo, Oslo, Norway; 4 Norwegian school of Health Sciences, Kristiania University College, Oslo, Norway; 5 KG Jebsen Centre for research on Influenza Vaccines, University of Oslo, Oslo, Norway; Johns Hopkins University, United States of America

## Abstract

Vaccination with naked DNA holds great promise but immunogenicity needs to be improved. DNA constructs encoding bivalent proteins that bind antigen-presenting cells (APC) for delivery of antigen have been shown to enhance T and B cell responses and protection in tumour challenge experiments. However, the mechanism for the increased potency remains to be determined. Here we have constructed DNA vaccines that express the fluorescent protein mCherry, a strategy which allowed tracking of vaccine proteins. Transfected muscle fibres in mice were visualized, and their relationship to infiltrating mononuclear cells could be determined. Interestingly, muscle fibers that produced MHC class II-specific dimeric vaccine proteins with mCherry were for weeks surrounded by a localized intense cellular infiltrate composed of CD45^+^, MHC class II^+^ and CD11b^+^ cells. Increasing numbers of eosinophils were observed among the infiltrating cells from day 7 after immunization. The local infiltrate surrounding mCherry^+^ muscle fibers was dependent on the MHC II-specificity of the vaccine proteins since the control, a non-targeted vaccine protein, failed to induce similar infiltrates. Chemokines measured on day 3 in immunized muscle indicate both a DNA effect and an electroporation effect. No influence of targeting was observed. These results contribute to our understanding for why targeted DNA vaccines have an improved immunogenicity.

## Introduction

Vaccination with naked DNA holds great promise for number of reasons such as ease of genetic construction, low cost, rapidity of mass production, high stability, and an attractive safety profile [1], [2]. However, the immunogenicity of plasmid DNA needs to be improved, and especially in larger animals and humans. Among major contributors to enhanced efficiency are vector design and optimization, and delivery methods such as gene gun [3], electroporation (EP) [4], [5], liposomes [6], nano particles [7], and viral capsids [1], [2], [8]. As concerns the immunogenicity-enhancing effect of EP, employed in the present study, it has been described that changing the field strength [9] or the field orientation (uni- or bipolar) [9] influence the number of transfected muscle fibres and thus the production of protein encoded by the transfected gene. Apart from enhancing transfection efficacy, electroporation may induce inflammatory infiltrates [10], [11], [12], and enhance production of proinflammatory cytokines [11]; factors that could contribute to potent immunoactivation.

A promising strategy to improve immune responses to protein antigen is to target antigen to antigen-presenting cells (APC). Given their exquisite specificity, antibodies are excellent for this purpose. In pioneering studies, antigens were chemically coupled to antibodies specific for APC; such antigen-antibody conjugates were shown to enhance immune responses [13], [14], [15]. Later, antigens have been genetically introduced into the C regions of recombinant antibodies engineered to express APC-specific variable regions [16], [17], [18], [19], [20], [21], [22], [23]. Antigens have been attached to the carboxy terminal tail of Fab fragments [16] or complete IgG [23], approaches which have certain limitations such as monovalency [16] and bulkiness [23]. In another strategy, 9–37 aa long antigenic peptides corresponding to T cell epitopes substitute loop regions between β-strands in the C-domain [17], [18], [22], [24]. The latter strategy has the advantage that the antibody structure is basically maintained with normal half lives *in vivo* [20]. The drawback is that the short antigenic peptides often lack conformational determinants and are poor at stimulating B cells and antibody responses. Moreover, the inserted peptides only fit to certain of the polymorphic MHC molecules in a species, which makes the T cell epitope insertion strategy less attractive as a general vaccine in outbred populations.

To overcome these limitations of previously recombinant antibodies, we generated a novel type of immunoglobulin-based molecules (vaccibodies) that are homodimers, targeting APC by single-chain variable fragment (scFv) specific for mouse MHC class II [25], CD40 [26] and TLR2 [27], or natural ligands like chemokines (MIP-1α and RANTES) [28], and deliver large genetically attached antigens with intact B-cell determinants [25], [28] for induction of potent T and B cell responses. Similar to complete antibodies [29], vaccibodies [25] can be delivered by injection of plasmid DNA into skeletal muscle followed by electroporation. Transfected muscle cells synthesize and secrete vaccibodies that can be found in serum and that are absorbed by APC [25]. Thus, antigen-primed APC as well as activation of CD4^+^ T cells can be demonstrated in draining lymph nodes, resulting in protective immunity against tumor challenges [25], [28].

However, the events taking place in the muscle with such DNA vaccines that encode APC-targeted proteins have not been studied. To address this issue we have here equipped the MHC II-specific vaccibodies with fluorescent mCherry, which makes the vaccine molecules easy to track. Fibers that produce MHCII-specific vaccine proteins become surrounded by infiltrating cells that stain positive for CD45, MHC class II, and CD11b. Large amounts of eosinophils were observed among the infiltrating cells from day 7. Chemokines measured on day 3 after treatment indicates an effect of both DNA-injection and electroporation.

## Materials and Methods

### Mice

BALB/c mice were bought from Taconic (Ry, Denmark), and were used for experiments between 6 and 10 weeks of age. The study was approved by the Norwegian Animal Research Committee (Oslo, Norway) (Permit number; Id-281 (2005), Id-666 (2007)).

### Constructions of mCherry vaccibody and mCherry-containing plasmids

Vaccibodies were constructed as explained by Fredriksen *et al*. [25]. The gene encoding mCherry [30] was kindly given to us by prof. Tsien (University of California, San Diego, CA). The mCherry gene was amplified from the pRSET-B vector by PCR, using primers introducing either 5′-BsmI, 3′-BamHI, or 5′-SfiI and 3′-SfiI restriction sites. The mCherry gen was cloned either into pLNOH_2_ between BsmI and BamHI or as antigenic unit in the vaccibody format between SfiI sites. In addition, a pLNOH_2_ plasmid expressing mCherry-His-Tag (for purification of native mCherry protein) was made, using a different 5′-BsmI primer. (Primers: restriction enzyme sites are underlined and the stop and start codons are indicated in bold. Gene codons of mCherry are depicted in capital letters. Introducing mCherry into pLNOH_2_ *5*′*-mCherry BsmI*; ggtgtgcattcc **atg** GTG AGC AAG GGC GAG GAG GAT AAC ATG *3*′*-mCherry BamHI*; ggtgggatcc **tca** CTT GTA CAG CTC GTC CAT GCC GCC G. Introducing mCherry with His-Tag into pLNOH_2_ *5*′*-His mCherry BsmI*; ggtgtgcattcc **atg** CGG GGT TCT CAT CAT CAT CAT CAT CAT GG. Cloning mCherry into antigenic site of ; *5*′*-mCherry SfiI*; ta ggcctcggtggcctg **atg** GTG AGC AAG GGC GAG GAG GAT AAC ATG. *3*′*-mCherry SfiI*; ta ggccctgcaggcc **tca** CTT GTA CAG CTC GTC CAT GCC GCC). mCherry had either targeting units specific for MHC class II (I-E^d^, scFv cloned from 14-4-4S B cell hybridoma) or were specific for the hapten 4-hydroxy-3-iodo-5-nitro-phenylacetyl (NIP scFv cloned from B1-8 hybridoma) [31]. These two mCherry containing vaccibodies are denoted αMHCII-mCherry and αNIP-mCherry, respectively.

### Transfections

Plasmid DNA encoding mCherry vaccibodies were transiently transfected into HEK293 cells by use of Lipofectamine 2000 (Invitrogen), using the protocol supplied by the manufacturer. Transfection efficiency of mCherry vaccibodies was monitored by Axiovert Leica microscopy with TRITC filter sets, and by testing supernatants in ELISAs.

### ELISA

ELISAs to evaluate the secretion of vaccibodies from transfected cells were performed using either an anti-mCherry antibody (clone 1 or clone 2 denoted a-mCherry.1 or a-mCherry.2) [32] or anti-human CH3 (clone A57H) or NIP-BSA (NIP conjugated to BSA) (1 µg/ml) for capture. As detection antibodies, either biotinylated anti-mCherry (a-mCherry.1-bio or a-mCherry.2-bio) [32] or biotinylated anti-human CH3 (a-hCH3-bio, clone HP6017) were used, followed by incubation with alkaline phosphatase-conjugated streptavidin and p-nitrophenyl phosphate substrate. For detection of anti-mCherry antibodies in sera of immunized mice, microtiter plates were coated with purified recombinant mCherry. Serum samples were serially diluted and incubated overnight, followed by incubation with alkaline phosphatase-conjugated anti-murine IgG (Fc-specific, cat. no. A2429, Sigma) or biotinylated IgG subclass-specific antibodies (anti-murine IgG1 (clone 10.9), anti-murine IgG2a (clone 8.3)). Threshold for end point titers was set to 2× the absorbance value of the negative controls (sera from mice given PBS).

### Gel electrophoresis

The samples were diluted in SDS-containing buffer and run on 4–20% Tris-Glycine polyacrylamide gels (Invitrogen). To reduce the disulfide bonds, some samples were treated with β-mercaptoethanol for 3 min at 95°C before electrophoresis. SeeBlue Plus2 Pre-Stained Standard (Invitrogen) was used to indicate the molecular weights. The proteins were blotted onto a polyvinyl fluoride membrane (Biorad) by 100 V for 1 h at 4°C. Milk and casein (5% and 1%, respectively) in 0.1% Tween 20 was used to block the membrane before incubation over night at 4°C with biotinylated anti-mCherry followed by incubation with streptavidin-conjugated HRP (Amersham Bioscience). The protein bands were developed by a chemiluminescent peroxidase substrate, Lumigen (GE Healthcare) and images were acquired by Kodak image station 2000R.

### Productions and purification of recombinant mCherry

pLNOH2 vector encoding mCherry with His-tag sequence expressed from a CMV promoter was stably transfected into NS0 cells by electroporation. Positive colonies were selected using Axiovert Leica microscopy with TRITC filter sets, and cloned by limiting dilution. Clones selected for strong fluorescence were expanded in roller bottles and grown for 7 days. Supernatants were harvested and mCherry proteins affinity purified on Ni-NTA superflow resin (Qiagen.) The eluted protein was dialysed against PBS 0.05% azid and stored as aliquots (1 mg/mL) −20°C.

### DNA injection and electroporation

BALB/c mice 6 to 10 weeks of age were immunized with DNA plasmids. Two different immunization methods were used in this study. (i) Animals immunized for measuring elicitation of anti-mCherry antibodies, or induction of chemokines in muscle, were DNA-injected and electroporated as follows: 6 to 8 weeks old BALB/c were anesthetized (Hypnorm Dormicum), their legs were shaved, and conductive gel was applied on the skin prior to injection of 50 µl of plasmids (purified with EndoFree plasmid kit (Qiagen); in concentration 1.0 µg/µl in 0.9% NaCl) in the femoral quadriceps muscle. Immediately after plasmid injection, electroporation was performed by use of the Elgen electroporator device equipped with a caliper electrode (Elgen, Inovio Biomedical Co., PA). The settings were: bipolar pulses of 100 mV ×0.2 ms with pulse sequence train and pulse sequence being 10 and 1000, respectively (10×1000-pulses protocol.) (ii) Animals immunized for *in vivo* microscopy and specimens for immunohistochemistry were immunized as follows: 6 to 8 weeks old BALB/c were anesthetized, soleus muscle was surgically exposed, and 10 µl of DNA solution (1.0 µg/µl in 0.9% NaCl) was injected into the centre of the muscle with a 701 Hamilton syringe (Hamilton, Reno, NV, USA). Subsequently, 5 trains of 1000 bipolar pulses (200 ms in each direction) with a peak-to-peak voltage of 10 V were run across the muscle, applied directly to the surface of the muscle by two silver electrodes (5×1000-pulses protocol). After treatment the dermis was sutured [33].

### Histopathology and immunohistochemistry

Mice were killed 7 days after DNA/EP immunization, and injected soleus muscle was excised and immediately frozen in OCT medium in isopropanol/nitrogen bath. Frozen muscles were cut into 5 µm sections, air dried, and fixed for 5 minutes in EtOH. Fixed sections were stained with hematoxylin and eosin (HE) before dehydration, mounting, and examination by microscopy. Sections for immunohistochemistry were air-dried before being packed in aluminium foil and frozen (−20°C). Slides were placed in a hydration chamber and 30% inactivated rat serum was added to sections for 1 hour, before biotinylated or FITC-conjugated antibodies were added for 1 hour, followed by streptavidin-Alexa488 or anti-fluorescein-Alexa488, respectively. Some slides were also stained with rabbit anti-laminin followed by anti-rabbit-Cy5. Primary antibodies used were anti-CD45-FITC (Clone 30-F11), anti-MHC class II-bio (TIB120 hybridoma, home made), anti-CD11b-FITC (Clone M1/70), and anti-laminin (cat. no. L9393, Sigma). Secondary antibodies used were Streptavidin-Alexa488 (cat. no. S-11223 Invitrogen), anti-fluorescein/Oregon Green-Alexa488 (cat. no. A-11096 Invitrogen), and anti-Rabbit-Cy5 (cat. no. ab6564, Abcam). Isotype controls used were Rat IgG_2a_-FITC (cat. no. 0117-02, SouthernBiotech), Rat IgG_2a_-FITC (cat. no. 0118-02, SouthernBiotech), Rat IgG_2b_-bio (cat. no. 0118-08, SouthernBiotech) and Hamster IgG_1_-bio (cat. no. 553951, BD Pharmingen).

### Microscopy and *in vivo* imaging

HE stained slides were examined and photographed on a Leica DMRB microscope with PL Fluotar oil-immersion objectives, connected to Leica DFC320 camera, and analyzed with Leica Image Manager and Photoshop CS4. Examination of immunohistochemistry slides and *in vivo* imaging were performed on a fixed-stage fluorescence microscope (Olympus BX50WI) with water-immersion objectives, adjustable halogen 12V (100W) lamp and non-adjustable quicksilver lamp. SIT camera (Hamamatsu C2400–08) coupled to an image processor (Hamamatsu ARGUS-20) and a Macintosh computer running Adobe Photoshop were used for photographs and analysis. *In vivo* imaging was performed on anesthetized animals by placing them on a heated plate and surgically opening the dermis to expose the muscle. Muscle was covered with Ringer-acetate, in some experiments including DAPI to visualize nuclei, and held in place with a coverslip mounted 2 mm above the muscle. The heated platform was placed on the microscope work platform and viewed through water-immersion objectives with long working distances. The lamp was operated at 3–5 V in order to avoid phototoxicity. For details see [34], [35], [36].

### Quantification of cytokines produced by plasmid-injected muscle

BALB/c mice were injected with plasmids encoding either αMHCII-mCherry or αNIP-mCherry (or PBS as negative control) into each quadriceps, followed by EP. Mice were sacrificed 3 or 21 days later, injected muscles were removed, and pooled for each animal. Muscles were washed in PBS, before adding approx. 1 ml RPMI1640 to a total volume of 1.5 ml, before disruption in an omni mixer. The homogenized solution was centrifuged, and supernatant collected and filtered trough a 0.45 µm HT Tuffryn membrane (PALL life science). To avoid repeated freeze-thaw cycles, 50 µl of each sample was aliquoted and kept at −70 °C until analysis. Cytokine and chemokine levels were measured using mouse-specific Bio-plex 23-plex assay (Biorad laboratories, Hercules, CA, US). Samples were adjusted to 0.5% BSA (Biotest, Dreieich, Germany), as were cytokine standards and background blanks. The assays were carried out according to the manufacturer's instructions. Measurements and data analysis were performed on Bio-Plex system, powered by xMAP technology by Luminex, operated with Bio-Plex Manager 4.1 software (BioRad Laboratories). The instrument was calibrated with the CAL2 settings (LOW RP1 target value) using Bio-Plex calibration beads (BioRad Laboratories). Samples were analyzed as single samples, whereas standards were analyzed in duplicates.

### Statistical analysis

All statistical analysis were carried out using GraphPad Prism, version 4 (GraphPad Software, San Diego, CA, USA). The different groups subjected to cytokine analyses were compared and analysed using the Mann-Whitney test. ELISA data were analysed by using two-tailed t-test. p<0.05 were considered statistically significant.

## Results

### Construction of mCherry-containing vaccibodies

**Figure 1:**
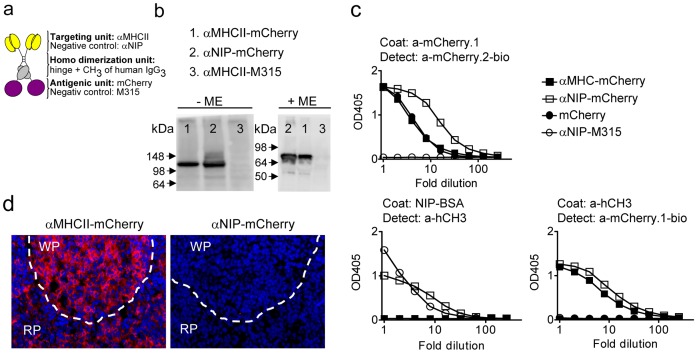
Characterization of mCherry-containing vaccibodies. (a) Schematic drawing of the vaccibody format [25] which consists of three functional units: a targeting unit, a dimerization unit (hinge h1 and h4 joined to the C_H_3 domain derived from human IgH chain γ3) followed by an antigenic unit. DNA encoding αMHCII or αNIP (non-targeted control) was inserted into the targeting unit, and DNA encoding mCherry or scFv of the myeloma protein M315 (negative control) was inserted into the antigenic unit. (b) The mCherry-containing vaccibodies form dimers. DNA encoding the vaccibodies was transfected into HEK293 cells, and the supernatants were either untreated (-ME) or treated with mercaptoethanol (+ME) prior to SDS-PAGE. Blotted proteins were detected by an anti-mCherry antibody [32]. (c) DNA encoding the vaccibodies or mCherry was transfected into HEK293 cells, and the supernatants were analyzed by ELISA as indicated. (d) αMHCII-mCherry but not αNIP-mCherry bound to sections of spleen from BALB/c. Stippled lines indicate the border between the red pulpa (RP) and the white pulpa (WP).

In order to track the vaccibodies as well as to identify their site of production, DNA encoding the fluorescent protein mCherry was inserted into the vaccibody format. mCherry was chosen because of favourable properties like short maturation time (15 minutes) which allows it to be visualized soon after translation; low pK_a_ (<4.5) which make it traceable after endocytosis; and good brightness and resistance to bleaching which are preferable properties in microscopy. In addition, the red shifted emission contributes to less disturbance of tissue auto fluorescence, and its monomeric structure and high tolerance for both N- and C-terminal fusions were attractive features for expression as part of vaccibody [37]. mCherry was genetically introduced into the antigenic unit of vaccibodies specific for either MHC class II (I-E) (denoted αMHCII) or the hapten 5-iodo-4-hydroxy-3-nitrophenacetyl (NIP) (denoted αNIP) (Figure 1a). Anti-NIP is anticipated not to recognize any molecules in mice, and was therefore used as non-targeted control.

### *In vitro* characterization of the vaccine molecules

To examine whether the vaccine molecules were secreted, Western blotting and ELISAs were performed on supernatants harvested from transfected HEK293 cells. Under non-reducing conditions, bands of approximately 140 kDa were detected while under reducing conditions bands of approximately 70 kDa were observed (Figure 1b). These sizes are consistent with predicted molecular weights and suggest that the mCherry-containing vaccibodies mainly are secreted as dimers. A battery of ELISAs verified that the vaccibodies were secreted from transfected HEK293 (Figure 1c). ELISAs confirmed that the non-targeted control, αNIP-mCherry, bound to NIP-conjugated BSA (Figure 1c). Moreover, the αMHCII-mCherry bound to spleen sections from BALB/c (Figure 1d) but not from C57BL/6 mice (data not shown), consistent with maintenance of I-E MHC II specificity. αNIP-mCherry did not bind spleen sections (Figure 1d). These results demonstrate that the two different targeting units maintained their specificity in the vaccibody format.

### The mCherry signal is associated with only a few nuclei in a muscle fiber

**Figure 2:**
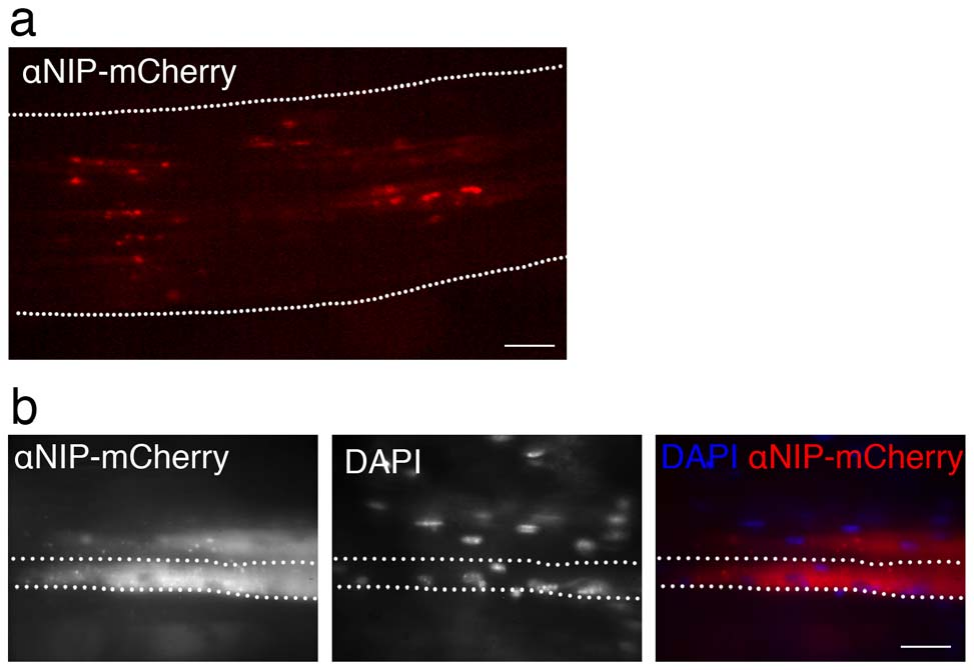
Muscle fibers transfected with αNIP-mCherry visualized *in vivo* in mice. DNA encoding αNIP-mCherry was injected into the soleus muscle followed by EP. One day later, the dermis was opened and mCherry (red) indicative of vaccibody expression was visualized by fluorescence microscopy of live muscle *in vivo* in anesthetized BALB/c mice. (a) Fluorescence microscopy of longitudinal, mid section of soleus muscle. The muscle belly boundary is indicated by dotted lines. Scale bar, 1 mm. (b) Fluorescence microscopy of two transfected muscle fibers. The dotted lines indicate the boundaries of one muscle fiber. DAPI was added to the muscle surface for staining of the nuclei. Scale bar, 50 µm.

We wanted to visualize the vaccine proteins in the muscles after DNA injection and electroporation (EP). In order to increase the control over the electroporation procedure, and to make it easier to localize the transfected muscle fibers, we did sight-guided DNA-injection and EP of muscle soleus, followed by suture. Before analysis by fluorescence microscopy, the mice were anesthetized, and the skin reopened to expose the muscle. Muscle fibers transfected with mCherry vaccibodies emitted fluorescence (Figure 2a). Nuclei were visualized by bathing the muscle during microscopy in Ringer-acetate solution containing DAPI (a fluorescent dye that binds DNA) (Figure 2b).

One muscle fiber/myocyte of muscle soleus, being approximately 7 mm long may contain around 400 nuclei [38]. Microscopy of live muscle showed strong mCherry signal juxtaposed to only one or a few nuclei in a long muscle fiber (Figure 2b, day 7 after DNA injection). mCherry signal was observed at the poles of nuclei, probably reflecting the Golgi apparatus, and in round structures within the muscle fiber. In general, there was a gradient of the mCherry signal in the fiber, being highest at the poles of the nuclei and waning with increased distance (Figure 2b).

### MHC II-targeted vaccibody enhances antibody responses and recruits immune cells

**Figure 3:**
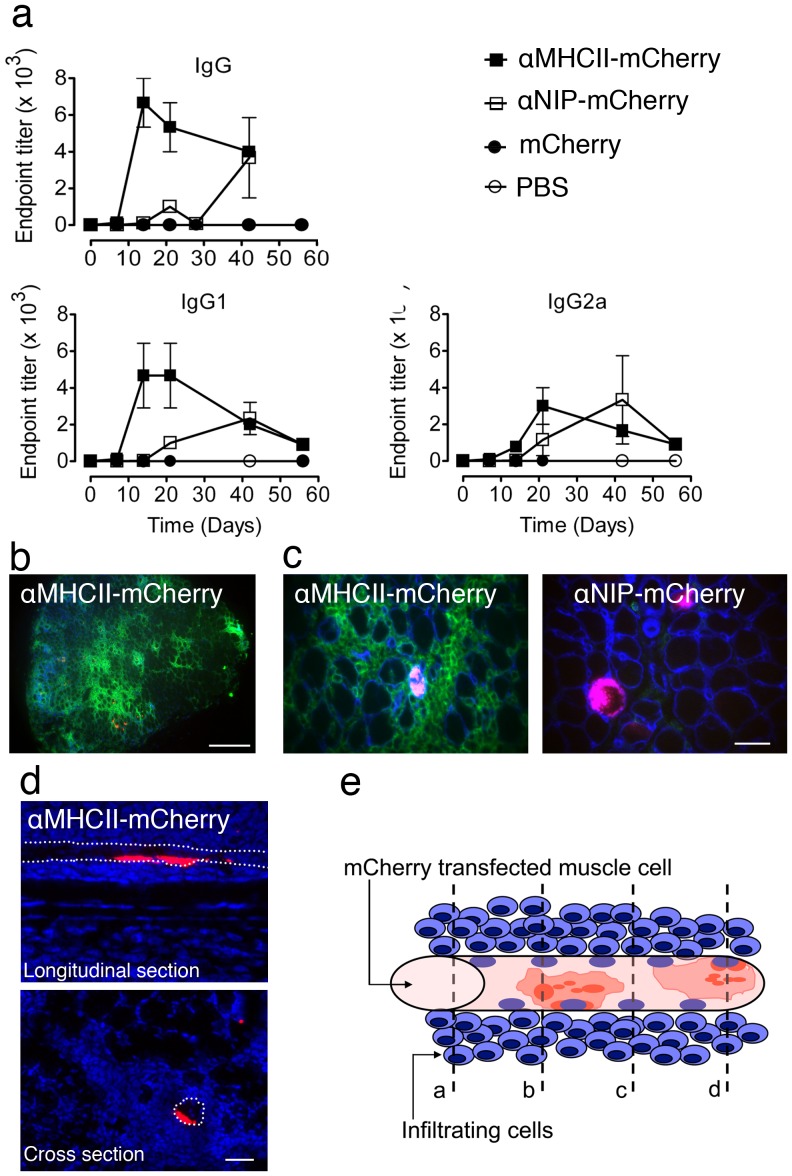
Immunization with αMHCII-mCherry followed by EP enhances antibody levels and cellular infiltrates in the muscle. (a) MHC II-targeting promotes antibody responses at early time points. PBS or DNA encoding mCherry or (either αMHCII-mCherry or αNIP-mCherry) was injected into quadriceps of BALB/c mice immediately followed by EP (day 0). Blood samples were harvested at the indicated time points. Serum antibody titers were determined by ELISA employing mCherry as coat. (Each symbol represents mean value of 2 or 3 mice, bars indicates ±SD.) Student's t-test of endpoint titers induced by αMHCII-mCherry in comparison to αNIP-mCherry gave *P*-values of 0.01 (IgG) and 0.04 (IgG_2a_) at day 14 (asterisk). (b) DNA encoding mCherry-containing vaccibodies was injected into the soleus muscle followed by EP. The muscle was harvested after 7 days, and cross sections of whole muscle were stained by use of anti-CD45 (clone 30-F11). Nuclei were stained by DAPI (blue). Scale bar, 1 mm. (c) DNA was given to BALB/c as described in *b*. After 7 days, soleus muscle was harvested and cross sections stained for CD45 (green) and laminin (blue). Red color in the left and right merged micrographs are indicative of αMHCII-mCherry and αNIP-mCherry, respectively. Scale bars, 50 µm. (d) Muscle harvested on day 7 from mice immunized with αMHCII-mCherry. A longitudinal and a cross section is shown in the top and bottom image, respectively. mCherry indicative of αMHCII-mCherry is shown in red and nuclei (DAPI-staining) in blue. White stippled lines indicate a muscle fiber/myocyte that expresses αMHCII-mCherry. Scale bar, 50 µm. (e) Interpretative drawing of a muscle fiber positive for mCherry (red color). The mCherry signal is concentrated around two nuclei (blue), and in puncta in the vicinity. Stippled lines in the drawing denoted a, b, c, and d, represent possible section levels. Some sections will contain both infiltrating cells and mCherry signal (b and d) while in others, only the infiltrating cells will be visible (a and c).

The vaccibody format was originally designed with the intent to target antigens to APCs, aiming to increase immune responses [25]. To examine whether the MHC II-targeted vaccibodies carrying mCherry in the antigenic unit induced higher levels of anti-mCherry antibodies, DNA encoding either αMHCII- or αNIP-mCherry was injected intramuscularly in quadriceps of BALB/c mice followed by EP. (Note that the electroporation procedure applied to the soleus and quadriceps muscles were different, see Materials and Methods.) Both types of vaccibodies induced antibody responses, however, the kinetics of the responses differed (Figure 3a). Anti-mCherry antibodies were detectable already at day 14 following immunization with αMHCII-mCherry. Antibodies following immunization with αNIP-mCherry appeared later but at day 40 the levels were similar to those induced by αMHCII-mCherry (Figure 3a).

In further experiments, we wanted to examine whether the difference in kinetics was reflected by local reactions in the muscle at the site of DNA injection. Soleus muscles from BALB/c mice immunized with αMHCII-mCherry were harvested after 7 days, cut into sections and stained with an antibody specific for CD45, which is expressed by cells of hematopoietic origin. Microscopy at low magnification revealed relatively few spots with mCherry signal, and an increased number of CD45^+^ cells present in focal areas throughout the muscle (Figure 3b and 3c, left image). To further identify the localization of the CD45^+^ cells, sections were immunostained with an antibody to laminin, a glycoprotein found in the basement membrane surrounding muscle fibers. The infiltrating CD45^+^ cells were localized outside of the basement membrane (Figure 3c, left image). Sections from muscles of BALB/c mice immunized with the non-targeted control (αNIP-mCherry) had comparatively few infiltrating CD45^+^ cells (Figure 3c, right image). Injection of the αMHCII-mCherry or αNIP-mCherry resulted in approximately the same number of mCherry-expressing muscle fibers (Figure 3c, and data not shown). Thus, these experiments suggest that the αMHCII-mCherry induces a focal inflammatory reaction at the injection site that depends on the targeting specificity.

To study the mCherry signal in more detail, we made longitudinal and cross sections of soleus muscle from mice immunized with MHC class II-targeted mCherry vaccibody (Figure 3d). Similar to the observation *in vivo* (Figure 2b), strong mCherry signal was found perinuclear to only a few nuclei within the length of a single muscle fiber, indicating a segmented expression of the mCherry vaccibodies within a fiber (Figure 3d). Thus, mCherry observed in a cross section most likely underestimate the number of transfected fibers, because a fiber that appears negative may nevertheless express mCherry at a level above or below the section, as illustrated in an interpretative drawing (Figure 3e). Nevertheless, in further experiments, when characterizing the infiltrates, we chose to investigate the immediate surroundings of fibers that expressed mCherry.

### Mononuclear and multilobulated cells are recruited following immunization with MHC class II-targeted vaccibodies

**Figure 4:**
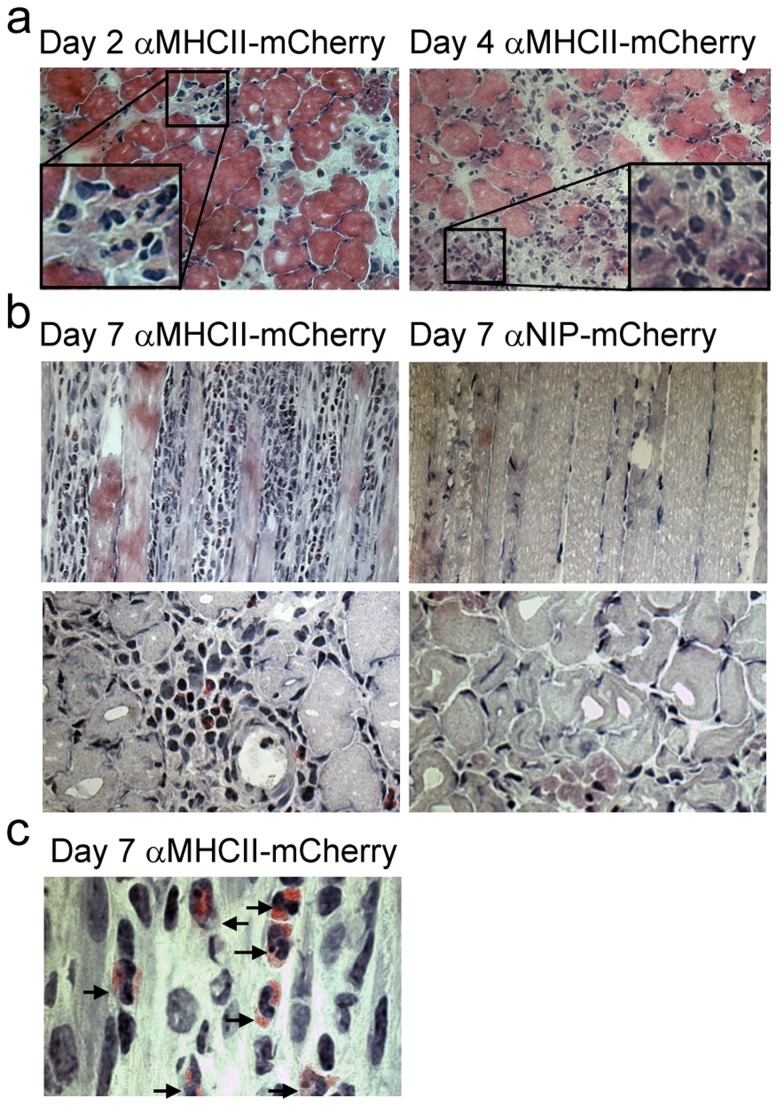
Immunization with αMHCII-mCherry leads to recruitment of mononuclear and multilobulated cells. DNA encoding αNIP-mCherry or αMHCII-mCherry was injected into the soleus muscle of BALB/c mice, followed by EP. Muscles were harvested at the indicated time points, flash-frozen and HE-stained. Representative images are shown. (a) Muscle injected with αMHCII-mCherry harvested on day 2 (left) and day 4 (right). Corner insets show high magnification of framed areas. (b) Comparison of muscle 7 days after immunization with αMHCII-mCherry or αNIP-mCherry. Longitudinal sections (upper row) and cross-sections (lower row) of muscles are shown. (c) Higher magnification of HE-stained section of soleus muscle harvested 7 days after immunization with αMHCII-mCherry. The infiltrate consisted of cells containing large nuclei, visible nucleoli, and sparse cytoplasm. In addition, we observed a large number of multilobulated cells with eosin-positive granula (arrows).

To characterize the CD45^+^ cellular infiltrate, muscle soleus was harvested 2, 4, and 7 days after immunization and stained with HE. αMHCII-mCherry induced cellular infiltrates present already 2 days after immunization. The infiltrating cells were localized between muscle fibers and the number increased with time (Figure 4a, b). The magnitude of the cellular infiltrate depended on MHC II-targeting since only few cells were observed between the fibers following delivery of αNIP-mCherry (Figure 4b, right panel). The increased cellular infiltration caused by αMHCII-mCherry, compared to αNIP-mCherry was also observed at day 13 (data not shown).

The infiltrate observed after DNA/EP immunization with αMHCII-mCherry, segregated the fibers and disrupted normal muscle morphology (Figure 4b, longitudinal section, left panel). At all time points, the infiltrate consisted of mononuclear cells as well as granulocyte-like cells with multilobulated nuclei (Figure 4a–c). Interestingly, by day 7, numerous cells with morphology similar to eosinophils (multilobulated nuclei and eosinophilic granula) were observed in the tissue (Figure 4c). The eosinophilic infiltrates depended on MHC class II-targeting, since there were no infiltrates of eosinophils following immunization with the αNIP-mCherry (Figure 4b, and data not shown).

### Characterization of the cellular infiltrate by immunostaining

**Figure 5:**
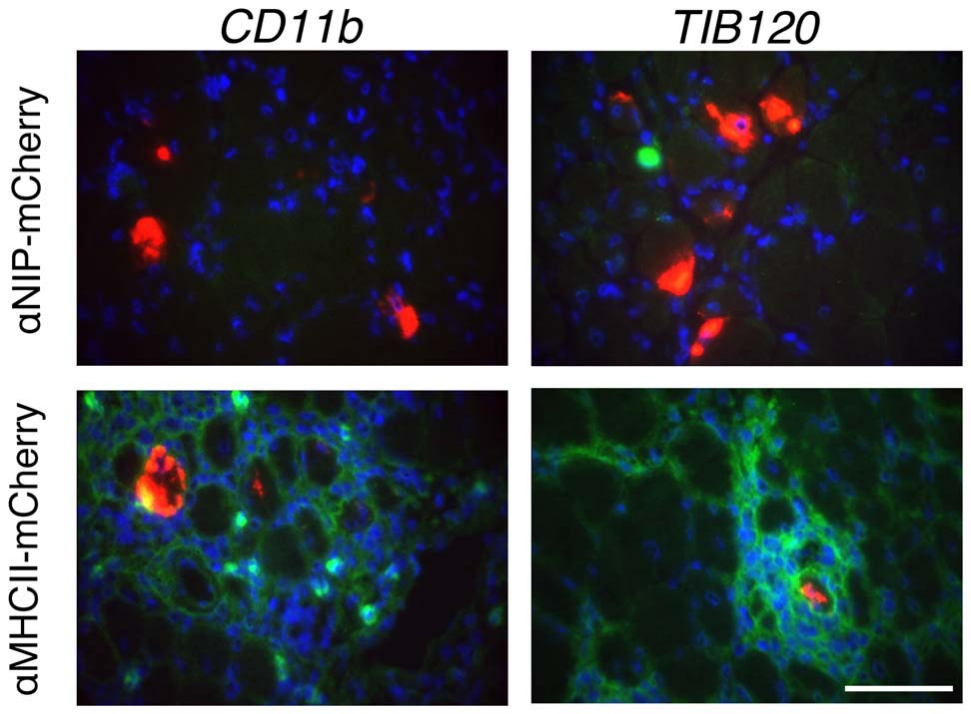
Characterization of infiltrating cells by immunostaining. The soleus muscle was harvested from BALB/c mice 7 days after injection of DNA encoding αNIP-mCherry or αMHCII-mCherry followed by EP. Immunostaining was performed with an antibody towards CD11b (clone M1/70) or MHC class II (clone TIB120). CD11b and MHCII are shown in green, nuclei in blue (DAPI), and mCherry in red indicative of αNIP-mCherry (upper row) or αMHCII-mCherry (bottom row). Scale bar, 80 µm.

αMHCII-mCherry or αNIP-mCherry DNA was delivered to the soleus muscle of BALB/c immediately followed by EP. The muscle was harvested at different time-points after immunization (day 3, 7, and 13, in altogether 8 experiments including a total of 30 mice). Immunostaining of muscles harvested 7 days post immunization, is presented in Figure 5. Similar to what we observed in previous experiments, focal infiltrates were only observed after immunization with the αMHCII-mCherry and not with the αNIP-mCherry (Figure 5). We stained sections for CD11b, a marker of monocytes/macrophages, DCs, neutrophils and natural killer cells. A major fraction of the cells had an intermediate signal for CD11b while a minor fraction of the cells were CD11b bright. In addition, we observed that most of the infiltrating cells were MHC II^+^ (TIB120).

### Cytokines in immunized muscle tissue

To examine whether there was a correlation between cellular infiltration and the cytokine milieu, we measured the levels of cytokines by use of Bio-Plex analyses of homogenized muscles from DNA/EP-immunized BALB/c. Because HE staining revealed an increasing number of infiltrating cells on days 2 and 4 after immunization with the targeted vaccine (Figure 4), we used muscle harvested on day 3 for the cytokine analysis.

**Figure 6:**
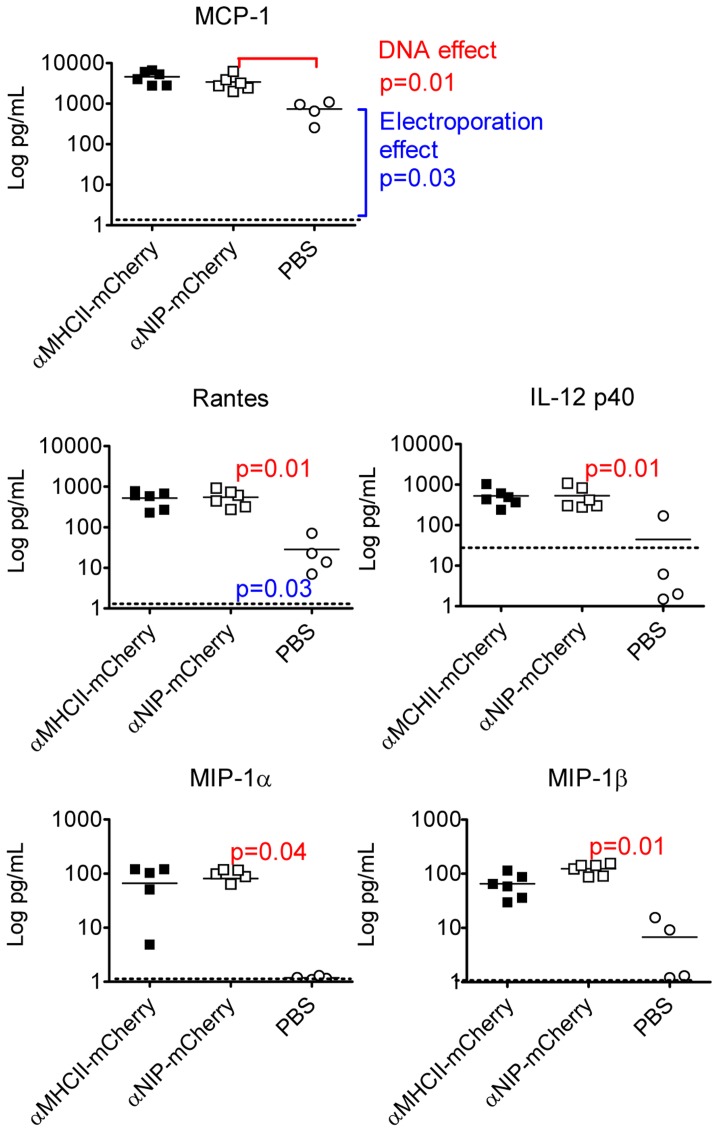
Cytokines in the muscle following intramuscular injection of DNA and EP. BALB/c were immunized in quadriceps with either PBS or DNA encoding αMHCII-mCherry or αNIP-mCherry, as indicated. After 3 days, muscles from DNA-injected (n = 6/group) and PBS-injected (n = 4/group) were harvested and homogenized before analysis by use of Bio-Plex. Baseline levels, indicated by stippled horizontal lines, represent values measured in muscle isolated 21 days after injection of PBS and electroporation (n = 4). Each symbol represents data from one mouse. Mean values are indicated by horizontal lines. Mann-Whitney test was used to calculate p-values.

Several observations could be made. First, injection of PBS/EP resulted in an increase in 13 out of 23 cytokines, when measured on day 3 compared to day 21 (Figure 6, Table I). Based on the assumption that values obtained on day 21 represent base line levels, our observations indicate that EP and/or the injection volume is a potent method for induction of several pro-inflammatory cytokines. Moreover, when compared to levels obtained by PBS/EP, injection of DNA/EP significantly boosted the expression of 5 out of the 13 cytokines (MCP-1, RANTES, IL-12p40, MIP-1α, MIP-1β). These data suggest that either DNA itself and/or produced vaccibody protein promoted expression of the 5 cytokines. The levels of 3 of the 5 cytokines, IL-12p40, MIP-1α and MIP-1β were enhanced following delivery of DNA, but not following PBS injection, suggesting that electroporation was not sufficient to enhance the levels of these 3 cytokines. Finally, none of the 23 cytokines that were measured showed significant differences after immunization with αMHCII-mCherry or αNIP-mCherry. The results are summarized in Table 1, which also includes a comparison with results published by others.

**Table 1:** Induction of cytokines by electroporation, and further enhancement by injection of DNA.

Study	EP induced cytokines	DNA enhancement of EP induced cytokines	Enhancement only by DNA/EP in combination
aThis study	IL-2, IL-3, IL-4, IL-5, IL-17, G-CSF, TNF-α, KC, MCP-1, Rantes	MCP-1, Rantes	IL-12p40, MIP-1α, MIP-1β
bPeng *et al*.[11]	MIP-1α, MIP-1β, MIP-1γ, IP-10, MCP-2, XCL-1		
d **Figarella** *et al*.[42]	TNF-α, IL-1β		

a 50 mg of DNA was injected in each quadriceps immediately followed by electroporation (Elgen, Inovio). 3 days later, 23 cytokines were measured in homogenized muscle (Bio-Rad, Bio-plex 23-assay). Cytokines not increased by either EP or DNA/EP were: IL-1α, IL-1β, IL-6, IL-9, IL-10, IL-12p70, IL-13, INF-γ, GM-CSF, and Eotaxin.

b Peng et al. stimulated tibialis anterior muscle in BALB/c mice with electroporation, and isolated muscle at different time points 2, 4 h and 1, 3d after stimulation. Isolated RNA and measured chemokines with cDNA array.

d Figarella et al. stimulated gluteus muscle with different combinations of electroporation, non-electroporation, DNA, PBS and empty DNA vector. TNF-α and IL-1β were measured using ELISA on samples isolated at 3 h.

## Discussion

In this study we show that introducing mCherry in the vaccibody format does not appreciably alter the antigenic or fluorescence properties of mCherry, neither does it reduce the specificity of the targeting unit for MHC class II. Furthermore, we show that transfected muscle cells emit mCherry fluorescence already one day post-immunization. Interestingly, by *in vivo* microscopy on DNA-injected and electroporated (EP) mice, we observed that only one or a few nuclei per transfected muscle fiber produced mCherry. mCherry accumulated in vesicle-like structures in proximity of apparently DNA-transfected nuclei. To what extent the segmented intracellular expression within the muscle fiber is reflected in concentration differences when vaccibodies are secreted to the extracellular fluid is unknown.

It is unclear whether mononuclear cells become transfected after DNA injection and electroporation of muscle [10], [39], [40]. One study [39] demonstrated that DNA injection into tibialis muscle of mice resulted in DNA uptake both by mononuclear cells and muscle cells. However, only the muscle cells were found to express the transgene, and the expression was enhanced by electroporation. Importantly, the uptake of DNA by mononuclear cells was not influenced by electroporation, and transfected mononuclear cells did not transcribe the transfected gene. Moreover, plasmid DNA was only found in vesicles, indicating endocytosis but not transfection. A later study [40] failed to detect transfected mononuclear cells positive for leucocyte surface markers located in muscle. Moreover, transgene-encoded mRNA was not detected in draining LN. In the most recent study [10], transfected mononuclear cells in the muscle were found to be satellite cells, staining positive for muscle-specific marker desmin, and negative for leucocyte specific surface markers CD3 and CD68. The above mentioned studies are consistent with the present study, since we have yet to find mCherry protein expressed in other cells than muscle fibers themselves, thus, we have no evidence of transfected APC. This observation is consistent with the finding that vaccibodies targeted to MHC-II increased anti-mCherry antibody responses over that seen with non-targeted vaccibody DNA. The targeting effect indicates that vaccibodies secreted by transfected muscle cells bind MHC class II molecules on APC for induction of immune responses. Stated otherwise, if transfection of mononuclear cells had been a major pathway for induction of enhanced immune responses upon DNA/EP immunization, no targeting effect would have been observed.

The electroporation procedure by itself is reported to induce inflammation [10], [11], production of chemokines and cytokines [11], and influx of a mixture of inflammatory cells [12], [40]. In addition, it has been described that injection of DNA, in absence of electroporation, induces upregulation of MCP-1 in muscle, and influx of IFN-γ secreting cells [41]. Although these studies differ in methodology and chemokines/cytokines measured (Table 1), there is an emerging picture that both electroporation and DNA injection can induce chemokine/cytokine secretion from muscle cells. Our results confirm and extend these previous findings by testing many more chemokines and cytokines. In addition, a novelty of our study is that DNA injection and electroporation appears to act in concert to improve expression of certain cytokines (IL-12p40, MIP-1α, MIP-1β). It may be noted that some cytokines (MIP-1α, RANTES, IL-8, MIG, IP-10, MIP-3α), overlapping with those described herein to be upregulated upon DNA/EP vaccination (Figure 6, Table 1), are also found to be increased in inflammatory diseases in muscle [42].

Interestingly, the different versions of vaccibodies, either αMHCII-mCherry or αNIP-mCherry, did not influence on the expression of cytokines in the muscle. This was surprising since the targeted and non-targeted vaccibodies differed in their abilities to induce antibodies and T cells (present study and [25]). These results indicate that the mechanism for enhanced immunogenicity of αMHCII-mCherry most probably must be sought in the draining lymph node. This suggestion is consistent with previous results demonstrating that αMHCII-vaccibody delivered as DNA/EP induces antigen-priming of APC and proliferation of CD4^+^ T cells in draining lymph nodes [25] (but not in spleen or non-draining lymph node). Moreover, we have recently found that B cell hybridomas are easily obtained by fusing cells from draining lymph nodes but not spleen of targeted vaccibody immunized mice, indicating that also B cell responses primarily take place in draining lymph nodes [32].

Even though the differences in immunogenicity between αMHCII-mCherry and αNIP-mCherry primarily appears to take place in the draining lymph node, clear cut differences were found in the immunized muscle for the two vaccibody versions. Thus, targeted vaccibodies induced more pronounced local infiltrates of macrophage-like CD45^+^MHCII^+^CD11b^+^cells, neutrophils and late-invading eosinophils. We failed to detect significant numbers of CD19^+^ B cells and CD11c^+^ dendritic cells (unpublished results). The latter preliminary observation appears to be at variance with a previous study [12] that described the mixed inflammatory infiltrate induced by DNA/EP to consist of CD19^+^ B-cells, CD3^+^CD4^+^ T-cells, CD11b^+^ macrophages and CD11c^+^ dendritic cells. The lack off infiltration of CD11c^+^ or CD19^+^ cells in the present study could by a result of methodology of DNA/EP immunization or a real absence. Further studies are clearly warranted to clarify this issue.

We have not been able to define a molecular mechanism for why targeted vaccibodies induced infiltrates, and why the infiltrates appeared to surround the αMHCII-mCherry producing fiber. In particular, we found no differences in levels of 23 cytokines in muscle, related to targeting or non-targeting of vaccibodies (see above). Perhaps subtle differences, unknown factors, or effects masked by the EP-induced inflammation, operate in the vicinity of muscle cells producing αMHCII-mCherry. It is tempting to speculate that ligation of MHC II on a few resident APC in muscle by αMHCII-mCherry, results in unknown chemoattractants being produced. However, as stated above, we have so far failed to observe binding of vaccibodies to MHCII^+^ APC in muscle.

Heredia *et al.* [43] has recently shown that eosinophils infiltrate cardiotoxin-injured muscle in mice and orchestrate, in an IL-4/IL-13 driven fashion, the differentiation of fibro/adipocyte progenitors (satellite cells) into myocytes required for complete regeneration of muscle tissue. In the present study, the late-appearing eosinophilic infiltrate is associated with αMHCII-mCherry. We postulate that this attraction of eosinophils is related to ligation of MHC class II on resident or infiltrating APC. We have failed to observe upregulation of eotaxin or IL-5, but hypothesize that existence a factor “X” could cause influx of eosinophils. It is well known that eosinophils are a major player of innate type 2 protection. It is therefore interesting to notice that the increase in antibodies caused by MHC-II targeting is mainly of the IgG1 subclass, which is known to be associated with Th2 responses. It is tempting to speculate that provision of MHCII ligation, *e.g.* by antibodies, can be used therapeutically in muscle diseases to induce infiltration of eosinophils and enhance muscle regeneration.

**Figure 7:**
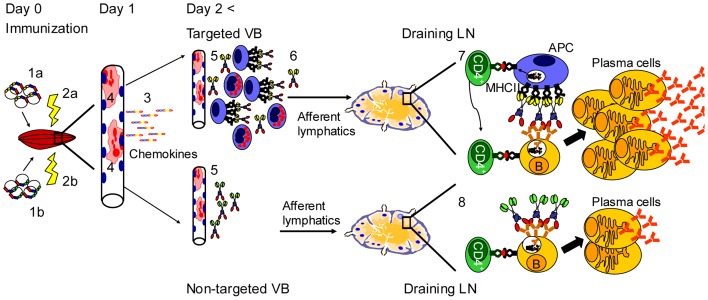
Interpretative drawing of proposed mechanism of action of targeted versus non-targeted DNA vaccines. Injection of DNA (*1a*,*1b*) and electroporation (*2a,2b*) have an adjuvant effect by inducing an inflammatory reaction in muscle resulting in elevated levels of chemokines (*3*). Transfected muscle cells produce (*4*, red colour) and secrete the targeted (*5, upper*) and non-targeted (*5, lower*) vaccine molecules. By an unknown mechanism, the targeted vaccibody induce an infiltration after >2 days predominantly composed of macrophages/DC, neutrophils and eosinophils (*6*). Targeted vaccibodies could drain to lymph node either unbound or bound to MHC II^+^ APC, but this remains to be determined. In contrast, non-targeted vaccibodies probably drain to lymph node as unbound molecules. In draining lymph nodes, targeted vaccibodies induce APC-B cell synapses and generation of T helper cells [25], resulting in strong stimulation of antigen specific B cells and generation of antibody secreting plasma cells (*7*). By contrast non-targeted vaccibodies do not induce APC-B cell synapses, and generation of T helper cells is poor [25] (*8*), resulting in inefficient plasma cell generation and antibody production.

In summary (Figure 7), the increased immunogenicity of MHCII targeted vaccine molecules for T cells [25], [44] and B cells is probably explained at the level of the draining lymph node. The transfected muscle cells primarily serve as production factories for secreted vaccine proteins. There are two non-exclusive explanations for how the vaccine molecules are transported from muscle to the draining lymph node. One possibility is that the vaccine molecules are transported as soluble molecules with afferent lymphatic vessels and bind to MHC II^+^ APC in the draining lymph node. Another possibility is that the targeted vaccine proteins bind the MHC II^+^ cells that accumulate in the vicinity of the transfected muscle cell. Such vaccibody-primed APC could then migrate to the draining lymph node where they could stimulate T and B cells. We have, however, thus far failed to detect that αMHCII-mCherry bind to MHC II^+^ infiltrating cells in muscle *in vivo*. This failure could be due to a too low sensitivity of fluorescence microscopy. Regardless of mechanism of transport, a fraction of the MHCII-specific vaccine molecules must have maintained intact conformation since stimulation of B cells is enhanced.

We postulate that MHC II-targeted vaccibodies is efficient at stimulating B cells due to formation of APC-B cell synapses in draining lymph nodes, as previously described *in vitro* [45]. MHCII-specific vaccine molecules could efficiently bridge APC and B cells. Bivalency of vaccibodies could contribute to synapse formation by increasing avidity for both APC and B cells (Figure 7). As concerns T cell help, MHC II-targeted vaccibody has previously been shown to increase antigen-priming of APC and enhance CD4^+^ T cell activation [25]. In addition, the B cells could via their BCR accumulate vaccine protein, process it, and present Ag-peptides on their MHCII molecules to CD4^+^ T cells (Figure 7). Thus, an APC-vaccibody-B cell synapse should concur with high levels of T cell help. As a consequence, B cells stimulated via its BCR by arrayed vaccine proteins in an APC-B cell synapse, and at the same time receiving strong T cell help, could efficiently develop into antibody secreting plasma cells (Figure 7). The combination of APC-targeting, bivalency of protein, and delivery as DNA, holds promise for establishment of a new generation of subunit vaccines.

## References

[1] Kutzler MA, Weiner DB (2008) DNA vaccines: ready for prime time? Nat Rev Genet 9: 776–788. 18781156 10.1038/nrg2432 PMC4317294

[2] Villarreal DO, Talbott KT, Choo DK, Shedlock DJ, Weiner DB (2013) Synthetic DNA vaccine strategies against persistent viral infections. Expert Rev Vaccines 12: 537–554. 23659301 10.1586/erv.13.33 PMC4317298

[3] Fynan EF, Webster RG, Fuller DH, Haynes JR, Santoro JC, et al (1993) DNA vaccines: protective immunizations by parenteral, mucosal, and gene-gun inoculations. Proc Natl Acad Sci U S A 90: 11478–11482. 8265577 10.1073/pnas.90.24.11478 PMC48007

[4] Aihara H, Miyazaki J (1998) Gene transfer into muscle by electroporation in vivo. Nat Biotechnol 16: 867–870. 9743122 10.1038/nbt0998-867

[5] Mathiesen I (1999) Electropermeabilization of skeletal muscle enhances gene transfer in vivo. Gene Ther 6: 508–514. 10476210 10.1038/sj.gt.3300847

[6] Pack DW, Hoffman AS, Pun S, Stayton PS (2005) Design and development of polymers for gene delivery. Nat Rev Drug Discov 4: 581–593. 16052241 10.1038/nrd1775

[7] Mastrobattista E, van der Aa MA, Hennink WE, Crommelin DJ (2006) Artificial viruses: a nanotechnological approach to gene delivery. Nat Rev Drug Discov 5: 115–121. 16521330 10.1038/nrd1960

[8] Fisher KJ, Jooss K, Alston J, Yang Y, Haecker SE, et al (1997) Recombinant adeno-associated virus for muscle directed gene therapy. Nat Med 3: 306–312. 9055858 10.1038/nm0397-306

[9] Gronevik E, von Steyern FV, Kalhovde JM, Tjelle TE, Mathiesen I (2005) Gene expression and immune response kinetics using electroporation-mediated DNA delivery to muscle. J Gene Med 7: 218–227. 15515140 10.1002/jgm.650

[10] Ratanamart J, Huggins CG, Shaw JA (2010) Transgene expression in mononuclear muscle cells not infiltrating inflammatory cells following intramuscular plasmid gene electrotransfer. J Gene Med 12: 377–384. 20373332 10.1002/jgm.1448

[11] Peng B, Zhao Y, Xu L, Xu Y (2007) Electric pulses applied prior to intramuscular DNA vaccination greatly improve the vaccine immunogenicity. Vaccine 25: 2064–2073. 17239494 10.1016/j.vaccine.2006.11.042

[12] Liu J, Kjeken R, Mathiesen I, Barouch DH (2008) Recruitment of antigen-presenting cells to the site of inoculation and augmentation of human immunodeficiency virus type 1 DNA vaccine immunogenicity by in vivo electroporation. J Virol 82: 5643–5649. 18353952 10.1128/JVI.02564-07 PMC2395223

[13] Kawamura H, Berzofsky JA (1986) Enhancement of antigenic potency in vitro and immunogenicity in vivo by coupling the antigen to anti-immunoglobulin. J Immunol 136: 58–65. 3079611

[14] Snider DP, Segal DM (1987) Targeted antigen presentation using crosslinked antibody heteroaggregates. J Immunol 139: 1609–1616. 2957430

[15] Casten LA, Pierce SK (1988) Receptor-mediated B cell antigen processing. Increased antigenicity of a globular protein covalently coupled to antibodies specific for B cell surface structures. J Immunol 140: 404–410. 2447176

[16] Baier G, Baier-Bitterlich G, Looney DJ, Altman A (1995) Immunogenic targeting of recombinant peptide vaccines to human antigen-presenting cells by chimeric anti-HLA-DR and anti-surface immunoglobulin D antibody Fab fragments in vitro. J Virol 69: 2357–2365. 7533857 10.1128/jvi.69.4.2357-2365.1995 PMC188908

[17] Lunde E, Bogen B, Sandlie I (1997) Immunoglobulin as a vehicle for foreign antigenic peptides immunogenic to T cells. Mol Immunol 34: 1167–1176. 9566764 10.1016/s0161-5890(97)00143-0

[18] Rasmussen IB, Lunde E, Michaelsen TE, Bogen B, Sandlie I (2001) The principle of delivery of T cell epitopes to antigen-presenting cells applied to peptides from influenza virus, ovalbumin, and hen egg lysozyme: implications for peptide vaccination. Proc Natl Acad Sci U S A 98: 10296–10301. 11517321 10.1073/pnas.181336898 PMC56955

[19] Lunde E, Western KH, Rasmussen IB, Sandlie I, Bogen B (2002) Efficient delivery of T cell epitopes to APC by use of MHC class II-specific Troybodies. J Immunol 168: 2154–2162. 11859101 10.4049/jimmunol.168.5.2154

[20] Schjetne KW, Thommesen JE, Fredriksen AB, Lunde E, Sandlie I, et al (2005) Induction of central T cell tolerance: recombinant antibodies deliver peptides for deletion of antigen-specific (CD4+)8+ thymocytes. Eur J Immunol 35: 3142–3152. 16184515 10.1002/eji.200425947

[21] Tunheim G, Schjetne KW, Rasmussen IB, Sollid LM, Sandlie I, et al (2008) Recombinant antibodies for delivery of antigen: a single loop between beta-strands in the constant region can accommodate long, complex and tandem T cell epitopes. Int Immunol 20: 295–306. 18252695 10.1093/intimm/dxm141

[22] Lunde E, Munthe LA, Vabo A, Sandlie I, Bogen B (1999) Antibodies engineered with IgD specificity efficiently deliver integrated T-cell epitopes for antigen presentation by B cells. Nat Biotechnol 17: 670–675. 10404160 10.1038/10883

[23] Bonifaz L, Bonnyay D, Mahnke K, Rivera M, Nussenzweig MC, et al (2002) Efficient targeting of protein antigen to the dendritic cell receptor DEC-205 in the steady state leads to antigen presentation on major histocompatibility complex class I products and peripheral CD8+ T cell tolerance. J Exp Med 196: 1627–1638. 12486105 10.1084/jem.20021598 PMC2196060

[24] Rasmussen IB, Oynebraten I, Hoydahl LS, Flobakk M, Lunde E, et al (2012) CD40/APC-specific antibodies with three T-cell epitopes loaded in the constant domains induce CD4+ T-cell responses. Protein Eng Des Sel 25: 89–96. 22233931 10.1093/protein/gzr063

[25] Fredriksen AB, Sandlie I, Bogen B (2006) DNA vaccines increase immunogenicity of idiotypic tumor antigen by targeting novel fusion proteins to antigen-presenting cells. Mol Ther 13: 776–785. 16414309 10.1016/j.ymthe.2005.10.019

[26] Schjetne KW, Fredriksen AB, Bogen B (2007) Delivery of antigen to CD40 induces protective immune responses against tumors. J Immunol 178: 4169–4176. 17371973 10.4049/jimmunol.178.7.4169

[27] Tunheim G, Thompson KM, Fredriksen AB, Espevik T, Schjetne KW, et al (2007) Human receptors of innate immunity (CD14, TLR2) are promising targets for novel recombinant immunoglobulin-based vaccine candidates. Vaccine 25: 4723–4734. 17499405 10.1016/j.vaccine.2007.04.004

[28] Fredriksen AB, Bogen B (2007) Chemokine-idiotype fusion DNA vaccines are potentiated by bivalency and xenogeneic sequences. Blood 110: 1797–1805. 17540847 10.1182/blood-2006-06-032938

[29] Tjelle TE, Corthay A, Lunde E, Sandlie I, Michaelsen TE, et al (2004) Monoclonal antibodies produced by muscle after plasmid injection and electroporation. Mol Ther 9: 328–336. 15006599 10.1016/j.ymthe.2003.12.007

[30] Shaner NC, Campbell RE, Steinbach PA, Giepmans BN, Palmer AE, et al (2004) Improved monomeric red, orange and yellow fluorescent proteins derived from Discosoma sp. red fluorescent protein. Nat Biotechnol 22: 1567–1572. 15558047 10.1038/nbt1037

[31] Neuberger MS, Rajewsky K (1981) Switch from hapten-specific immunoglobulin M to immunoglobulin D secretion in a hybrid mouse cell line. Proc Natl Acad Sci U S A 78: 1138–1142. 6940132 10.1073/pnas.78.2.1138 PMC319962

[32] Oynebraten I, Lovas TO, Thompson K, Bogen B (2012) Generation of antibody-producing hybridomas following one single immunization with a targeted DNA vaccine. Scand J Immunol 75: 379–388. 21955209 10.1111/j.1365-3083.2011.02639.x PMC3417379

[33] Rana ZA, Ekmark M, Gundersen K (2004) Coexpression after electroporation of plasmid mixtures into muscle in vivo. Acta Physiol Scand 181: 233–238. 15180796 10.1111/j.1365-201X.2004.01282.x

[34] Bruusgaard JC, Liestol K, Ekmark M, Kollstad K, Gundersen K (2003) Number and spatial distribution of nuclei in the muscle fibres of normal mice studied in vivo. J Physiol 551: 467–478. 12813146 10.1113/jphysiol.2003.045328 PMC2343230

[35] Bruusgaard JC, Brack AS, Hughes SM, Gundersen K (2005) Muscle hypertrophy induced by the Ski protein: cyto-architecture and ultrastructure. Acta Physiol Scand 185: 141–149. 16168008 10.1111/j.1365-201X.2005.01462.x

[36] Bruusgaard JC, Gundersen K (2008) In vivo time-lapse microscopy reveals no loss of murine myonuclei during weeks of muscle atrophy. J Clin Invest 118: 1450–1457. 18317591 10.1172/JCI34022 PMC2262032

[37] Shaner NC, Steinbach PA, Tsien RY (2005) A guide to choosing fluorescent proteins. Nat Methods 2: 905–909. 16299475 10.1038/nmeth819

[38] Bruusgaard JC, Liestol K, Gundersen K (2006) Distribution of myonuclei and microtubules in live muscle fibers of young, middle-aged, and old mice. J Appl Physiol 100: 2024–2030. 16497845 10.1152/japplphysiol.00913.2005

[39] Dupuis M, Denis-Mize K, Woo C, Goldbeck C, Selby MJ, et al (2000) Distribution of DNA vaccines determines their immunogenicity after intramuscular injection in mice. J Immunol 165: 2850–2858. 10946318 10.4049/jimmunol.165.5.2850

[40] Gronevik E, Tollefsen S, Sikkeland LI, Haug T, Tjelle TE, et al (2003) DNA transfection of mononuclear cells in muscle tissue. J Gene Med 5: 909–917. 14533200 10.1002/jgm.416

[41] Stan AC, Casares S, Brumeanu TD, Klinman DM, Bona CA (2001) CpG motifs of DNA vaccines induce the expression of chemokines and MHC class II molecules on myocytes. Eur J Immunol 31: 301–310. 11265647 10.1002/1521-4141(200101)31:1<301::AID-IMMU301>3.0.CO;2-K

[42] Figarella-Branger D, Civatte M, Bartoli C, Pellissier JF (2003) Cytokines, chemokines, and cell adhesion molecules in inflammatory myopathies. Muscle Nerve 28: 659–682. 14639580 10.1002/mus.10462

[43] Heredia JE, Mukundan L, Chen FM, Mueller AA, Deo RC, et al (2013) Type 2 innate signals stimulate fibro/adipogenic progenitors to facilitate muscle regeneration. Cell 153: 376–388. 23582327 10.1016/j.cell.2013.02.053 PMC3663598

[44] Grodeland G, Mjaaland S, Roux KH, Fredriksen AB, Bogen B (2013) DNA Vaccine that Targets Hemagglutinin to MHC Class II Molecules Rapidly Induces Antibody-Mediated Protection against Influenza. J Immunol 191: 3221–3231. 23956431 10.4049/jimmunol.1300504 PMC3767367

[45] Batista FD, Iber D, Neuberger MS (2001) B cells acquire antigen from target cells after synapse formation. Nature 411: 489–494. 11373683 10.1038/35078099

